# Geographical Variation in Health-Related Quality of Life Among Older US Adults, 1997–2010

**DOI:** 10.5888/pcd11.140023

**Published:** 2014-07-03

**Authors:** Diana Kachan, Stacey L. Tannenbaum, Henry A. Olano, William G. LeBlanc, Laura A. McClure, David J. Lee

**Affiliations:** Author Affiliations: Stacey L. Tannenbaum, Henry A. Olano, William G. LeBlanc, Laura A. McClure, David J. Lee, University of Miami Miller School of Medicine, Miami, Florida.

## Abstract

**Introduction:**

Health-related quality of life (HRQOL) is an important predictor of morbidity and mortality; however, its geographical variation in older adults in the United States has not been characterized. We compared HRQOL among older adults in the 50 US states and the District of Columbia using the Health and Activities Limitation Index (HALex). We also compared the HRQOL of 4 regions: South, West, Midwest, and Northeast.

**Methods:**

We analyzed pooled data from 1997 through 2010 from the National Health Interview Survey for participants aged 65 or older. HALex scores (which range from 0 to 1.00, with higher values indicating better health) were calculated by combining data on participants’ perceived health and activity limitations. We ranked states by mean HALex score and performed multivariable logistic regression analyses to compare low scores (defined as scores in the lowest quintile) among US regions after adjustment for sociodemographics, health behaviors, and survey design.

**Results:**

Older residents of Alaska, Alabama, Arkansas, Mississippi, and West Virginia had the lowest mean HALex scores (range, 0.62–0.68); residents of Arizona, Delaware, Nevada, New Hampshire, and Vermont had the highest mean scores (range, 0.78–0.79). Residents in the Northeast (odds ratio [OR], 0.66; 95% confidence interval [CI], 0.57–0.76) and the Midwest (OR, 64; 95% CI, 0.56–0.73) were less likely than residents in the South to have scores in the lowest quintile after adjustment for sociodemographics, health behaviors, and survey design.

**Conclusion:**

Significant regional differences exist in HRQOL of older Americans. Future research could provide policy makers with information on improving HRQOL of older Americans.

## Introduction

Health-related quality of life (HRQOL) is predictive of mortality in older adults (1), and it is a newly measurable target for improvement in *Healthy People 2020* (2). The HRQOL of some older adults, such as those who have arthritis, has been characterized (3), and an association between the HRQOL of older adult population groups and health factors such as physical activity (4,5) and obesity (6,7) has been established.

Studies show a regional variation in the health behaviors of older adults. For example, 1 study found state variations in smoking, drinking, and physical activity levels among older adults (8). A study of Medicare beneficiaries in 2002 showed a regional variation in the functional status of older adults, with those residing in the southern US states reporting greater functional limitations (9). Regional variation in obesity status has also been recognized (10). Older adults living in southern states were found to have the lowest healthy life expectancy (11). Nationally representative data were used to examine geographical variation in the self-rated health of older US adults (12). For the general population, an HRQOL measure derived from the number of healthy days was used to examine state variation in HRQOL (13). However, geographic variation in HRQOL of older adults has not been examined. The objective of our study was to use a nationally representative data set and the Health and Activities Limitation Index (HALex) to compare HRQOL among adults aged 65 or older in the 50 states and the District of Columbia. We also compared HRQOL for 4 regions of the United States: West, Midwest, South, and Northeast (14).

## Methods

We pooled data from 1997 through 2010 from the National Health Interview Survey (NHIS), an annual multistage probability household survey of the US civilian noninstitutionalized population. We included participants aged 65 or older (n = 79,863, representing approximately 34,587,284 people). HALex scores were calculated according to methods described by Livingston and Ko (14); scores range from 0 to 1.00, with lower scores reflecting poorer HRQOL. Calculation of HALex scores is based on participants’ responses to questions about their health and activity limitations, including the following: needing help with personal care and routine needs, being unable to work because of a health problem, being limited in the kind or amount of work that the participant is able to perform, and being limited in any other way.

We ranked states by calculated mean HALex score and sorted states by mean score. We used multivariable logistic regression to compare low HALex scores (defined as scores in the lowest quintile) among US regions (South, West, Midwest, and Northeast); we used the South as the reference group. States were assigned to regions according to the 2010 US Census classification (15) (Figure). Regression analyses were adjusted for the following sociodemographic variables: as continuous variables, age and body mass index; and as categorical variables, sex, race/ethnicity (non-Hispanic black, non-Hispanic white, Hispanic, or other), education (less than a high school degree, high school degree or equivalent, or more than a high school degree), health insurance status (has insurance or does not have insurance), employment status (employed or unemployed), smoking and drinking history (current, former, or never for each), and compliance with the American College of Sports Medicine and the American Heart Association recommendations for physical activity (yes or no) (16). Compliance is defined as performing moderate physical activity for 30 minutes or more per day for 5 or more days per week or vigorous physical activity for 20 minutes or more per day for 3 or more days per week (16). Sampling weights were used to adjust for survey design (17). All analyses were conducted in SAS version 9.2 (SAS Institute Inc, Cary, North Carolina). Because state data were not publically available, analyses were performed at the National Center for Health Statistics Research Data Center in Hyattsville, Maryland, by a member of the research team (W.G.L.). This study was approved by the University of Miami Institutional Review Board.

**Figure:**
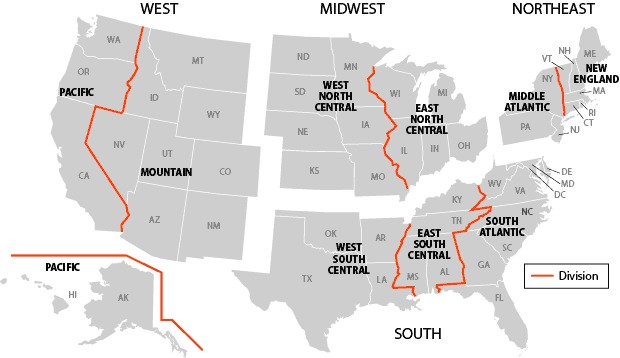
States were grouped into West, Midwest, Northeast, and South, according to US Census regions and divisions (15).

## Results

The mean HALex score for older US adults was 0.735 (standard error, 0.001, Table 1). Residents of Alaska, Alabama, Arkansas, Mississippi, and West Virginia had the lowest mean HALex scores (range, 0.62–0.68), whereas residents of Arizona, Delaware, Nevada, New Hampshire, and Vermont had the highest scores (range, 0.78–0.79) (Table 1). Residents of the Northeast (odds ratio [OR], 0.66; 95% confidence interval [CI], 0.57–0.76) and Midwest (OR, 0.64; 95% CI, 0.56–0.73) were less likely than residents of the South to be in the lowest quintile of HALex scores after adjustment for sociodemographics, health behaviors, and survey design (Table 2)

**Table 1:** Mean Health and Activities Limitation Index^a^ Scores, by State, Participants Aged 65 or Older, National Health Interview Survey, 1997–2010

State	Sample Size	Estimated Population	Mean Score (SE)	Rank
All states	79,863	34,587,284	0.735 (0.001)	NA
Alabama	1,533	662,542	0.657 (0.009)	50
Alaska	73	38,676	0.616 (0.049)	51
Arizona	1,427	646,305	0.779 (0.010)	4
Arkansas	965	432,278	0.680 (0.019)	47
California	8,343	3,265,104	0.733 (0.004)	31
Colorado	926	391,420	0.766 (0.008)	10
Connecticut	1,036	467,428	0.754 (0.010)	15
District of Columbia	204	71,562	0.715 (0.024)	37
Delaware	218	109,028	0.788 (0.018)	1
Florida	6,155	2,579,298	0.756 (0.005)	14
Georgia	1,863	818,102	0.721 (0.008)	35
Hawaii	478	198,790	0.752 (0.009)	16
Idaho	308	141,044	0.728 (0.011)	33
Illinois	3,349	1,483,862	0.749 (0.005)	18
Indiana	1,696	743,281	0.744 (0.010)	24
Iowa	872	386,691	0.760 (0.011)	12
Kansas	793	332,663	0.740 (0.013)	26
Kentucky	1,148	523,258	0.686 (0.007)	45
Louisiana	1,189	508,999	0.708 (0.010)	42
Maine	458	223,202	0.749 (0.011)	19
Maryland	1,253	590,046	0.748 (0.008)	21
Massachusetts	1,843	850,085	0.772 (0.008)	9
Michigan	2,775	1,222,078	0.734 (0.007)	30
Minnesota	1,236	572,982	0.772 (0.006)	8
Mississippi	828	363,832	0.669 (0.019)	48
Missouri	1,766	789,941	0.705 (0.009)	43
Montana	306	124,147	0.730 (0.022)	32
Nebraska	613	280,481	0.772 (0.018)	7
Nevada	473	211,611	0.787 (0.010)	3
New Hampshire	320	162,612	0.788 (0.017)	2
New Jersey	2,467	1,121,428	0.751 (0.005)	17
New Mexico	767	237,822	0.715 (0.013)	38
New York	5,449	2,205,410	0.749 (0.005)	20
North Carolina	2,303	1,050,880	0.708 (0.007)	41
North Dakota	263	107,990	0.738 (0.022)	27
Ohio	3,327	1,538,785	0.720 (0.006)	36
Oklahoma	1,031	457,966	0.708 (0.019)	40
Oregon	992	445,257	0.744 (0.013)	25
Pennsylvania	3,762	1,797,310	0.746 (0.006)	23
Rhode Island	261	136,223	0.763 (0.020)	11
South Carolina	1,223	567,687	0.715 (0.008)	39
South Dakota	293	139,407	0.735 (0.024)	29
Tennessee	1,515	670,593	0.681 (0.010)	46
Texas	5,192	1,881,478	0.704 (0.005)	44
Utah	522	268,451	0.737 (0.010)	28
Vermont	148	70,843	0.777 (0.014)	5
Virginia	1,991	929,455	0.728 (0.012)	34
Washington	1,438	648,596	0.758 (0.008)	13
West Virginia	572	265,964	0.663 (0.020)	49
Wisconsin	1,737	781,984	0.775 (0.005)	6
Wyoming	163	72,406	0.747 (0.023)	22

Abbreviations: SE, standard error; NA, not applicable.

a Scores range from 0 to 1.00, with higher values indicating better health (14).

**Table 2:** Likelihood of Being in the Lowest Quintile of HALex^a^ Scores, Participants Aged 65 Years or Older, National Health Interview Survey, 1997–2010

Covariate	Odds Ratio (95% Confidence Interval)	*P* Value
**Age**	1.05 (1.04–1.05)	<.001
**Body mass index**	1.03 (1.02–1.04)	<.001
**Female vs male**	1.14 (1.02–1.27)	.026
**Uninsured vs insured**	0.99 (0.56–1.74)	.96
**Employed vs unemployed**	0.07 (0.04–0.14)	<.001
**Race**		
Black vs non-Hispanic white	1.24 (1.09–1.42)	.002
Hispanic vs non-Hispanic white	1.21 (1.02–1.43)	.03
Other vs non-Hispanic white	1.22 (0.91–1.64)	.19
**Education**		
High school degree vs more than high school degree	1.08 (0.93–1.24)	.31
Less than high school degree vs more than high school degree	1.65 (1.46–1.87)	<.001
**Smoking status**		
Current smoker vs nonsmoker	1.59 (1.34–1.89)	<.001
Former smoker vs nonsmoker	1.46 (1.30–1.65)	<.001
**Use of alcohol**		
Current drinker vs nondrinker	0.32 (0.28–0.38)	<.001
Former drinker vs nondrinker	1.01 (0.90–1.14)	.88
**Does not meet physical activity recommendations of *Healthy People 2010* vs does meet recommendations**	9.56 (7.20–12.68)	<.001
**Region**		
West vs South	0.92 (0.79–1.08)	.32
Midwest vs South	0.64 (0.56–0.73)	<.001
Northeast vs South	0.66 (0.57–0.76)	<.001

a Health and Activities Limitations Index (14).

## Discussion

We found variation in HRQOL among regions and states. Scores ranged from 0.62 (Alaska) to 0.79 (Delaware), the former score representing on average an inability to perform a major activity (such as an activity of daily living) and the latter representing a partial limitation in a nonessential activity (18). Older adults in the South were more likely to have the lowest HRQOL scores than were their Northeast and Midwest counterparts even after adjustment for sociodemographic factors and health behaviors. State ranking by mean HALex score showed a similar pattern: most of the lowest-ranking states were located in the South. Studies show that older adults in southern states have high rates of disability, mobility issues, and functional limitations (9,19,20) as well as risk factors for poor functional status (diabetes [21], stroke [22,23], obesity [10], and hypertension [24]). Higher disability rates persist among older adults who migrate from the South to other regions (20,25). Better prevention of disability risk factors in the general population of the South could help improve HRQOL of older adults in that region.

The pattern of low HALex scores in the South and higher scores in other regions is not totally consistent. For example, Delaware (included as a southern state in this study) ranked the highest of all states, and Florida ranked 14th, far above all other southern states. In other studies, Florida showed evidence of not being similar to other southern states — older residents of Florida have a higher life expectancy (11), a lower prevalence of smoking, and a higher prevalence of compliance with daily physical activity recommendations (8) than older residents of other southern states. Older adults in Delaware are also among the most likely to meet the daily requirements for physical activity and have a higher life expectancy than residents of other southern states (except Florida). Alaska ranked 51st in our study. Older residents of Alaska have the highest prevalence of risky drinking of all states; however, their life expectancy is comparable with the life expectancy for residents of the West and Midwest (8,11).

Among the potential explanations for the inconsistent rankings of Delaware and Alaska is the HALex calculation. The index includes measures of functional limitations, and therefore it depends on the individual’s physical limitations as well as the individual’s access to transportation, services, and assistive devices. HALex tends to give more biased (ie, lower) measures for people with disabilities than some other HRQOL scores (26,27). A study of Medicare beneficiaries suggested that a higher prevalence of functional limitations among older women in the South could be attributed to areas of lower population density with higher poverty levels (9). Although Delaware and Alaska are among the top 15 states for highest median household income (28), Delaware has the 6th-highest population density in the United States and an award-winning public transportation system complete with a state-wide door-to-door paratransit service for the elderly (29,30). Alaska has the lowest population density in the United States and a public transportation system that is not easily accessible (29). Although differences in HALex scores between states can be partially explained by the relative ease of getting around and differences in the levels of physical activity and alcohol consumption, further research is necessary to examine other potential causes of such differences.

Our state rankings differed from a previous Behavioral Risk Factor Surveillance System (BRFSS)–based study of the general adult population (13), especially for the highest HRQOL scores; however, rankings were similar for the lowest HRQOL scores. The BRFSS study used the Healthy Days measure to derive a utility-based index similar to HALex, and the authors noted that the validity of such derivation has not been determined. Our study used a validated HRQOL measure that was created for use with NHIS data, and therefore it is a more valid representation of the actual HRQOL of the study participants. In addition, some of the inconsistencies in state rankings between the general adult BRFSS study and our older adult study may result from differences in perceptions of perfect health between participants of different age groups and from differences in accommodations accessible to adults of different ages (such as services available through Medicare or state-funded services for older adults). These differences would have affected the HALex measurements of self-rated health and functional limitations. Finally, such differences could have resulted from the limitations of the data. NHIS is not designed as a state survey, and BRFSS has low response rates. Findings from both data sources might complement each other in assessing population needs in future studies.

Significant regional variation exists in the HRQOL of older individuals in the United States. Measures aimed at improving the HRQOL of older adults are warranted in southern states and in Alaska.
